# Essential Oils and Extracts of *Juniperus macrocarpa* Sm. and *Juniperus oxycedrus* L.: Comparative Phytochemical Composition and Anti-Proliferative and Antioxidant Activities

**DOI:** 10.3390/plants11081025

**Published:** 2022-04-09

**Authors:** Luciano Meringolo, Marco Bonesi, Vincenzo Sicari, Simone Rovito, Nicodemo Giuseppe Passalacqua, Monica Rosa Loizzo, Rosa Tundis

**Affiliations:** 1 Department of Pharmacy, Health and Nutritional Sciences, University of Calabria, 87036 Rende, Italy; luciano2793@gmail.com (L.M.); marco.bonesi@unical.it (M.B.); monica_rosa.loizzo@unical.it (M.R.L.); 2 Department of Agricultural Science, Mediterranean University of Reggio Calabria, 89123 Reggio Calabria, Italy; vincenzo.sicari@unirc.it; 3 Museum of Natural History of Calabria and Botanic Garden, University of Calabria, 87036 Rende, Italy; simone.rovito@unical.it (S.R.); nicodemo.passalacqua@unical.it (N.G.P.)

**Keywords:** *Juniperus*, phytochemicals, chemotaxonomy, antioxidant activity, anti-proliferative activity

## Abstract

In this work, we conducted a comparative phytochemical, chemotaxonomic, and biological study of essential oils (EOs) and extracts (ethyl acetate and methanol) obtained from the leaves of *Juniperus* *macrocarpa* and *J. oxycedrus*. The dominant compounds of *J. macrocarpa* EO, analysed by gas chromatography (GC) and gas chromatography-mass spectrometry (GC-MS), are α-pinene, sabinene, manoyl oxide, and germacrene D, whereas α-pinene, limonene, (*Z*,*E*)-farnesol, β-pinene, and γ-cadinene are the most representative volatiles of *J. oxycedrus* EOs. A multivariate analysis of EOs, included a selection of literature data comparing our samples to samples of *J. oxycedrus*/*macrocarpa*/*deltoides* from the Mediterranean area, was performed. As evident by high-performance liquid chromatography (HPLC) analyses, apigenin, (−)-epicatechin, and luteolin were abundant in *J. oxycedrus* extracts, while gallic acid, kaempferol-3-*O*-glucoside, and protocatechuic acid were the dominant constituents of *J. macrocarpa* extracts. EOs and extracts have been investigated for their potential antioxidant properties and anti-proliferative activity against lung adenocarcinoma (A549), breast cancer (MCF-7 and MDA-MB-231), and lung large cell carcinoma (COR-L23) human cell lines. The methanol and ethyl acetate extracts of *J. oxycedrus* exerted the most valuable antioxidant activity and exhibited the most promising activity against the COR-L23 cell line with an IC_50_ of 26.0 and 39.1 μg/mL, respectively, lower than that obtained with the positive control (IC_50_ of 45.5 μg/mL). To the best of our knowledge, this is the first report highlighting the anti-proliferative activity of *J. oxycedrus* and *J. macrocarpa* extracts against this lung cancer cell line. Our results indicate that *J. oxycedrus* may be considered a source of natural compounds with antioxidant and anti-proliferative effects that could be suitable for future applications.

## 1. Introduction

**Figure 1:**
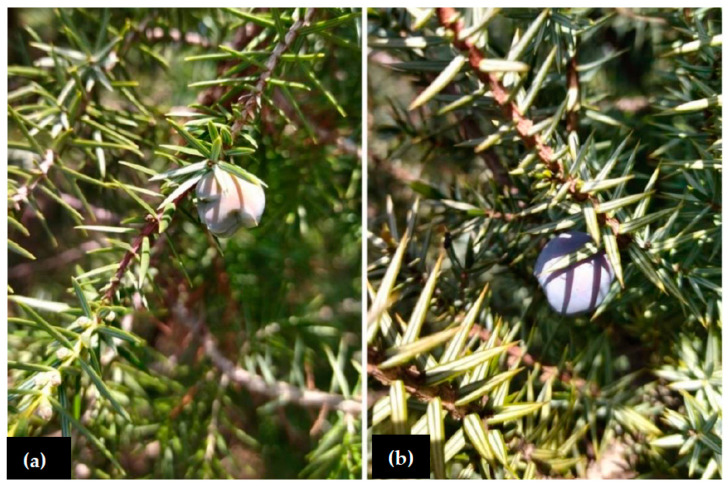
(**a**) *Juniperus oxycedrus* L. and (**b**) *J. macrocarpa* Sm.

The genus *Juniperus* (Cupressaceae) is traditionally divided into three sections: J. sect. Caryocedrus Endl., J. sect. *Juniperus*, and J. sect. Sabina Spach [1]. The *Juniperus oxycedrus* group is included within J. sect. *Juniperus* [1] and includes two chemotaxonomically and phylogenetically close Mediterranean species: *Juniperus oxycedrus* L. and *J. macrocarpa* Sm. (Figure 1), with the latter often treated as a subspecies of the former. A third species included in this group, *J. deltoids* Adams, is hardly morphologically discernible from *J. oxycedrus*, but some molecular evidence suggests a late Miocene divergence [2]. Considering these features, we referred to *Juniperus oxycedrus* s.l. when the literature data were not sufficient to define an exact species.

*Juniperus oxycedrus* L. (prickly juniper, plum juniper, cade juniper, red-berry juniper, cada) is a shrub or small tree native to the western Mediterranean region, from Morocco and Portugal eastward to southern Italy, growing on a variety of rocky sites from sea level up to 1600 m altitude. *J. macrocarpa* (maritime juniper), which is morphologically [3], molecularly [2], and genetically [4] differentiated from *J. oxycedrus*, is a Mediterranean species growing on coastal sand dunes. *Juniperus* false fruits, female cones—improperly called ‘‘berries’’—are used as a spice, mainly in European cuisine; they are used in Northern European and particularly Scandinavian cuisine to impart a sharp, clear flavour to meat dishes [5]. *J. oxycedrus* s.l. berries have widely been used in traditional medicine for the treatment of gastrointestinal disorders, diabetes, common colds, as expectorant in cough, to treat calcinosis in joints, as diuretic to pass kidney stones, against urinary inflammations, haemorrhoids, and as hypoglycaemic; leaves and berries are applied externally for parasitic disease [5,6,7,8,9].

A literature survey found many studies that analysed the composition of the essential oils (EOs) from berries and leaves of *Juniperus* species and their bioactivities, mainly antibacterial, antiviral, antifungal, and antioxidant properties [3,7,10,11,12,13,14,15,16,17,18,19,20,21]. These EOs are used for their biological activity but also in the food and cosmetic industries.

Less investigated are the polar extracts. Among literature studies, some investigations on *J. oxycedrus* revealed the presence of polyphenols such as biflavones, flavonols, and coumarins and antioxidant, hypoglycaemic, anti-inflammatory, antimicrobial, analgesic, anti-nociceptive, and antifungal activities [5,6,22,23,24,25].

Cancer is one of the most important health problems of our community, and natural compounds are considered valuable candidates for the development of new intervention approaches to improve the therapeutic index and address the frequent occurrence of chemo-resistance to current anti-cancer therapies.

Over the last decades, scientific progress has highlighted the prominent role of reactive oxygen species (ROS) in the pathogenesis of inflammatory diseases, neurodegenerative diseases, and cancer [26]. Particularly, in the early stages of cancer, excessive ROS production and related oxidative stress are considered as important molecular hallmarks. Hence, the initial stages of carcinogenesis could be suppressed by antioxidants.

In spite of the extensive studies on the bioactivities of *Juniperus* EOs, the anti-proliferative activity of polar extracts is still less investigated. For this reason, as part of ongoing work to investigate medicinal plants as sources of antioxidant and anti-proliferative agents [27,28], the present study aimed to examine the anti-proliferative effects against human lung adenocarcinoma (A549), human breast cancer ER+ (MCF-7), triple negative breast adenocarcinoma (MDA-MB-231), and human lung large cell carcinoma (COR-L23) cell lines and the antioxidant activity of EOs and polar extracts from two *Juniperus* species with globally recognized health benefits from Italy, *J. macrocarpa* and *J. oxycedrus*, in relation to their chemical profiles analysed by gas chromatography (GC), gas chromatography-mass spectrometry (GC-MS), and high-performance liquid chromatography-diode-array detector (HPLC-DAD).

The main goal of our research was to support the use of these *Juniperus* species as valuable sources of bioactive compounds with potential benefits as anti-proliferative agents.

## 2. Results

### 2.1. Chemical Composition of Essential Oils

*J. macrocarpa* and *J. oxycedrus* fresh leaves were subjected to hydro-distillation and exhaustive maceration by using two solvents at different polarities such as ethyl acetate and methanol.

Essential oils were obtained with yields of 0.2 and 0.3% for *J. macrocarpa* and *J. oxycedrus*, respectively. Analysing polar extracts, the highest extraction yield was obtained by using methanol with yields of 10.4 and 10.8% for *J. macrocarpa* and *J. oxycedrus*, respectively. Yields of 6.1 and 4.2% for *J. macrocarpa* and *J. oxycedrus*, respectively, were obtained by using ethyl acetate as solvent for maceration.

The essential oils of *J. macrocarpa* and *J. oxycedrus* were analysed by GC and GC-MS. Table 1 reports the main identified compounds listed in order of elution on an HP5 MS column with their retention index, identification methods, and percentage contribution.

Forty-seven constituents (accounting for 90.2% of the total composition of the essential oil) were tentatively identified in the essential oil of *J. macrocarpa* in which the dominant compounds were monoterpene hydrocarbons (57.8%), followed by diterpenes (11.9%) and sesquiterpene hydrocarbons (8.7%). α-Pinene (25.3%), sabinene (8.2%), and manoyl oxide (6.6%) were the most abundant compounds.

Forty-four volatiles (accounting for 97.0% of the total composition of the essential oil) were identified in the essential oil of *J. oxycedrus*. α-Pinene (36.9%), limonene (6.5%), (*Z*,*E*)-farnesol (6.3%), and β-pinene (5.5%) were the most representative constituents. As for *J. macrocarpa*, the dominant class of constituents was represented by monoterpene hydrocarbons (57.5%). Sabinene was identified in the essential oil of *J. macrocarpa* but not in *J. oxycredus*. The amounts of α-pinene, β-pinene, limonene, γ-cadinene, δ-cadinene, and caryophyllene oxide detected in *J. macrocarpa* were lower than those characterizing the essential oil of *J. oxycredus*. Conversely, higher amounts of *p*-cymene, (*E*)-β-ocimene, terpinolene, α-terpineol, manoyl oxide, and germacrene D were found in *J. macrocarpa*.

**Figure 2:**
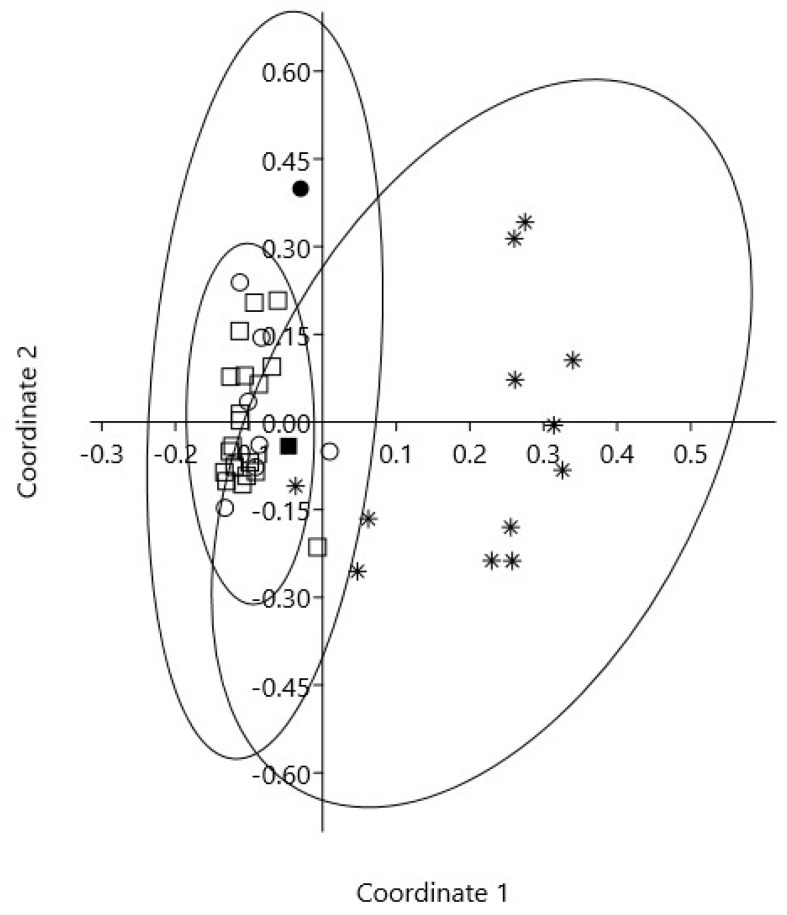
Scatterplot of the first and second axes of principal coordinate analysis (PCoA) of essential oils of *J. oxycedrus* (squares), *J. macrocarpa* (circles), and *J. deltoides* (asterisk). Filled symbols represent our samples. Species’ 95% concentration ellipses are superimposed.

A multivariate analysis of essential oils included a selection of literature data comparing our samples to samples of *J. oxycedrus/macrocarpa/deltoides* from the Mediterranean area was performed. On the first two axes of principal coordinate analysis (PCoA, Figure 2) (explaining 71.9 and 8.9% of variability), *J. deltoides* was separated from the other taxa on the first axis, which showed most of the variability explained (71.9%).

The analysis of *J. oxycedrus* and *J. macrocarpa* essential oils resulted in largely overlapping sections on the left side of the scatterplot, with no way to distinguish them.

Our *J. oxycedrus* sample was inside the variability of the species, whereas our sample of *J. macrocarpa* was shown to be differentiated from the *J. oxycedrus*/*J. macrocarpa* group on the second axis. However, the second axis, which explained 8.9% of the variability, did not reflect any particular taxonomic pattern, and it was linked to the variability of oils such as manoyl-oxide, abietatriene, and abieta-7,13-diene, which do not discriminate among taxa (*p* > 0.1).

**Figure 3:**
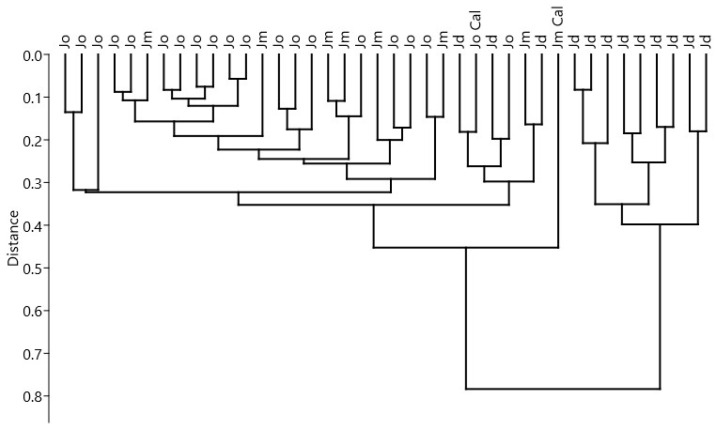
Dendrogram of cluster analysis of essential oils of *J. oxycedrus* (Jo), *J. macrocarpa* (Jm), and *J. deltoides* (Jd). “Cal” represents our samples.

Cluster analysis confirmed the best grouping number of two (Figure 3), which was also significant (silhouette average width = 0.75). The first cluster was formed by *J. deltoides* samples, the second by mainly *J. oxycedrus*/*J. macrocarpa* samples. The second cluster showed the first subcluster with our *J. macrocarpa* sample well-separated from the others and a second subcluster, which included all *J. deltoides* samples that were not included in the first cluster together with our *J. oxycedrus* sample.

In the third subcluster, all other *J. oxycedrus*/*J. macrocarpa* samples were included with no differentiation between the species.

Among selected essential oil constituents, limonene was the one most characteristic for *J. deltoides* (22.22 ± 6.8), and β-pinene for the *J. oxycedrus*/*J. macrocarpa* group. Higher content in sabinene and manoyl oxide, together with lower content in β-caryophyllene, distinguished the Calabrian *J. macrocarpa* sample from the *J. oxycedrus*/*J. macrocarpa* group.

### 2.2. The Chemical Profiles of Juniperus Polar Extracts

*J. macrocarpa* and *J. oxycedrus* were extracted by maceration using two solvents with different polarities. With the hypothesis that the constituents of these species could be efficiently extracted using solvents with different polarities, we evaluated the efficiency of ethyl acetate and methanol in the recovery of phytochemicals from *Juniperus* extracts.

These extracts were analysed by applying HPLC-DAD. Data are reported in Table 2. Based on literature data, we chose apigenin, caffeic acid, (+)-catechin, chlorogenic acid, (−)-epicatechin, gallic acid, kaempferol, kaempferol-3-*O*-glucoside, luteolin, neochlorogenic acid, protocatechuic acid, quercetin, quercetin-3-*O*-glucoside, rutin, syringic acid, and vanillic acid as markers.

*J. oxycedrus* and *J. macrocarpa* showed similar flavonoid and phenolic compound fingerprints, whereas they differed in terms of quantitative content. In light of the obtained data, the major identified constituents belonging to the flavonoid class were (−)-epicatechin, rutin, catechin, quercetin, quercetin-3-*O*-glucoside, and luteolin.

(−)-Epicatechin was present in *J. oxycedrus* in quantities 10–20 times greater than in *J. macrocarpa*. Quercetin-3-*O*-glucoside was also present in significantly great content, mainly in *J. oxycedrus* ethyl acetate extract (2937.2 μg/g), compared to that in *J. macrocarpa* (1533.6 μg/g).

### 2.3. Antioxidant and Anti-Proliferative Properties

*Juniperus* species were investigated herein for their antioxidant and anti-proliferative properties. Cancer is a global challenge with a high impact on human health, causing morbidity and mortality. Although important advances have been obtained in early cancer diagnosis and cancer treatment, there is still a need for new compounds that contribute to and improve the therapeutic approaches in actual use.

Natural compounds represent a countless source of new molecules that can be used as anti-cancer agents once their activity, bioavailability, and toxicity are demonstrated to be acceptable. The ability of many natural compounds to act also as antioxidant agents could enhance their anti-cancer activity. In fact, several studies have demonstrated that antioxidant activities such as free radical scavenging, lipid peroxidation, and metal chelating activities from natural extracts can enhance the anti-cancer properties of many anti-cancer drugs [29,30].

The study of antioxidant properties, mainly of antioxidant agents that are multifunctional or mixtures that act in complex systems, cannot be adequately evaluated by a simple antioxidant assay without due regard for the many variables that may influence the results. Several procedures may be required to assess such antioxidant effects. For these reasons, the potential antioxidant activities of *Juniperus* species were analysed by using four in vitro assays, namely the 2,2’-azino-bis(3-ethylbenzothiazoline-6-sulfonic acid (ABTS), 2,2-diphenyl-1-picrylhydrazyl (DPPH), ferric reducing antioxidant power (FRAP), and β-carotene bleaching tests. A concentration–response relationship was observed for all tested samples. The IC_50_ values are given in Table 3.

Generally, *J. oxycedrus* was more active than *J. macrocarpa*. In particular, the ethyl acetate and methanol extracts exhibited promising ABTS radical scavenging activity with IC_50_ values of 9.3 and 6.2 μg/mL, respectively.

Lower radical scavenging activity was observed in the DPPH tests, where IC_50_ values of 19.7 and 20.6 μg/mL, for ethyl acetate and methanol, respectively, were found. Moreover, the ethyl acetate extract was shown to possess the ability to protect lipids from peroxidation, as evidenced by the β-carotene bleaching assay (IC_50_ values of 15.1 and 13.2 μg/mL at 30 and 60 min of incubation, respectively).

Among *J. macrocarpa* samples, the methanol extract demonstrated the strongest ABTS and DPPH radical scavenging activity with IC_50_ values of 39.1 and 29.3 μg/mL, respectively. The most polar extract also exhibited an antioxidant capacity in the β-carotene-linoleic acid test system with IC_50_ values of 65.1 and 62.5 μg/mL after 30 and 60 min of incubation, respectively. Both essential oils were able to exert ferric reducing activity with FRAP values of 2.4 and 3.8 μM Fe(II)/g for *J. macrocarpa* and *J. oxycedrus*, respectively, when tested at 2.5 mg/mL.

*J. macrocarpa* and *J. oxycedrus* extracts and essential oils elicited concentration-dependent inhibition of the cellular viability of four human cancer cell lines such as MCF-7, MDA-MB-231, A539, and COR-L23 cells. IC_50_ values are reported in Table 4.

The COR-L23 lung cancer cell line was the most sensitive to *J. oxycedrus* extracts, with IC_50_ values of 26.05 and 39.12 μg/mL for methanol and ethyl acetate extract, respectively. Both values were lower than that obtained with positive control vinblastine sulfate salt (IC_50_ value of 45.5 μg/mL).

Lung cancer is the leading cause of about 18.6% of total cancer deaths worldwide. Non-small cell lung cancer (NSCLC) and small cell lung cancer (SCLC) are the two major groups of lung cancer based on a histological classification. NSCLC represents 80% of all lung cancer cases and is subdivided into adenocarcinoma, large cell carcinoma, and squamous cell carcinoma. The management of the treatment of lung cancer is often difficult due to the development of drug resistance, non-specific targeting of the anti-cancer drugs, and/or drug–drug interactions. Therefore, the search for new and active compounds is necessary.

*J. oxycedrus* methanol extract was the only extract able to inhibit proliferation of human lung adenocarcinoma cell line (A549), with an IC_50_ value of 87.9 μg/mL.

Against breast cancer cell lines (MCF-7 and MDA-MB-231), the most active sample was the EO of *J. macrocarpa*, with IC_50_ values of 85.4 and 96.4 μg/mL for MCF-7 and MDA-MB-231, respectively.

*J. oxycedrus* EO was inactive against all tested cell lines.

## 3. Discussion

In this work, a comparative phytochemical, chemotaxonomic, and biological study of essential oils (EOs) and extracts (ethyl acetate and methanol) obtained from the leaves of *Juniperus macrocarpa* and *J. oxycedrus* was conducted.

Numerous studies are present in the literature on the chemical composition of *J. macrocarpa* EOs (Table 5). The content of α-pinene in the *J. macrocarpa* EO analysed in our work was in agreement with that found by Valentini et al. [18], who reported α-pinene content of 22.8% in a sample collected in Puglia (southern Italy). The same authors also analysed *J. macrocarpa* aerial parts from the Abruzzo hills (central Italy) in two different months, emphasizing how the plant harvesting period, as well as the altitude at which it grew, could affect the content of active ingredients.

Both samples collected in Abruzzo were rich in α-pinene, showing high percentages in the range 73.5–81.3%. Although similar in α-pinene content to the sample analysed by Valentini et al. [18], the Apulian oil showed some peculiarities especially regarding the identification of 1,8-cineole (9.1%), a compound not found in our sample, and α-terpineol (18.7%), a compound identified in our oil with a lower percentage of 2.9%. Interestingly, the Abruzzo *J. macrocarpa* oils revealed the presence of the sesquiterpene γ-muurolene (2.6%), which was not identified in either the Apulian oil or in our sample, suggesting that this sesquiterpene is characteristic of *J. macrocarpa* grown in the hills rather than at sea level. On the other hand, our *J. macrocarpa* essential oil was richer in *p*-cymene (13.2%) and sabinene (8.2%) compared with the oil samples analysed by Valentini et al. [18].

The chemical variability of *J. macrocarpa* essential oils from Italy is evident also in other countries such as Greece, Tunisia and Turkey [7,16,17,19]. The Greek essential oils showed α-pinene and cedrol as the dominant compounds, both differing in content by about double depending on whether they came from southern or south-eastern Greece. Voulrioti-Araopi et al. [19], analysing the essential oil of *J. macrocarpa* collected in Athens (south-eastern Greece), showed an α-pinene content of 58.0%, a value about double that described by Stassi et al. [17].

The cedrol content (13.9%) reported for the essential oil from southern Greece [17] was also double compared to that reported by Voulrioti-Arapi et al. [19] (7.3%).

Medini et al. [16] analysed the EOs extracted from two samples of *J. macrocarpa* collected in north and central Tunisia (Laazib and Hawaria, respectively), not highlighting a notable difference in their phytochemical profiles, with α-pinene (15.9–22.8%) and sabinene (9.1–12.1%) as major compounds. Sezik et al. [7], who analysed three samples of EOs extracted from the leaves of *J. macrocarpa* collected in Turkey in three months (May, August, and October), confirmed the existence of seasonal variability in the phytochemical profile of this plant species. In particular, the authors highlighted that manoyl oxide (7.7–21.9%) was the main compound, followed by α-pinene (7.2–11.1%) and cedrol (2.3–9.7%). Except for α-pinene with the highest percentage (11.1%) in October, the highest values were observed in samples harvested in August.

Characteristic were the phytochemical profiles of *J. macrocarpa* oils from Croatia and Algeria [12,15]. In detail, the essential oil reported by Lesjak et al. [15] showed α-pinene (49.4%) and germacrene D (18.1%) as the main components, both present at very high percentages compared to the values shown in Table 5 in reference to the sample analysed by us, followed by β-phellandrene (3.8%), present in lower percentages in the samples from other countries and absent from the Italian samples. Djebaili et al. [12] highlighted germacrene D as the main component of the oil from Algeria, with a percentage of 21.3%, much higher than that found in the oil analysed by us.

Among the main components in the EOs from Spain, Adams et al. [9] highlighted α-pinene (22.6%), with a content very similar to the oils from Italy, and terpinen-4-ol (7.3%), the latter showing content clearly higher than that in the oil analysed by us. Sabinene showed the highest percentage content of 26.5%, a value about three times higher than that reported by us. Sabinene is a characteristic component of *J. macrocarpa* that is not found in *J. oxycedrus*, a peculiarity also observed by Valentini et al. [18].

From analysis of Table 5, it is also evident that there are other clear variations in the phytochemical profiles of these two plants. A first fundamental difference concerns the presence of different typical compounds in one or the other species. Eight compounds—sabinene, tricyclene, *p*-mentha-1,5-dien-8-ol, verbenone, (−)-carvone, piperitone, (−)-bornyl acetate, and β-cubebene—were exclusively present in *J. macrocarpa*. On the other hand, four compounds not found in *J. macrocarpa* were identified as distinctive of *J. oxycedrus*: myrtenal, α-gurjunene, *trans*-α-bergamotene, and (*Z*)-phythol. Besides these compounds, other interesting differences in the content of other constituents can be highlighted. Indeed, *p*-cymene showed a content about 13 times higher in *J. macrocarpa* compared to that in *J. oxycedrus*. The contents of α-terpineol, (*E*)-β-ocimene, manoyl oxide, and α-phellandrene in *J. macrocarpa* were also very high, each showing values about three times higher than in *J. oxycedrus*.

Conversely, *J. oxycedrus* showed higher content of monocyclic and bicyclic monoterpenes, including limonene, α-pinene, and β-pinene, as well as sesquiterpenes such as farnesol, trans-caryophyllene, caryophyllene oxide, δ-cadinene, and γ-cadinene. This high α-pinene content in *J. oxycedrus* from Italy was highlighted also by Valentini et al. [18], who found the percentage of α-pinene to be 26.3%. The Abruzzo essential oil showed a high limonene content (30.0% vs. 6.3% in our sample).

Limonene and α-pinene were also the main components in the *J. oxycedrus* essential oils.

Adams et al. [9] analysed and compared the phytochemical profiles of the essential oils extracted from the leaves of four *J. oxycedrus* samples, two of which were collected in Greece (north and south) and two in Spain. The oils were dominated by α-pinene and limonene, with moderate amounts of β-pinene, myrcene, *p*-cymene, β-phellandrene, and manoyl oxide. Adams et al. [9] evidenced a variability in the composition, especially in relation to the amounts of α-pinene, limonene, and δ-3-carene. In particular, the oil from southern Greece was found to be richer in α-pinene (42.7%) than that from northern Greece (25.3%), while the oils from Spain were found to be very similar, with α-pinene content of just over 40%. Both Spanish oils and southern Greek oils had content of this monoterpene very similar to that found by us (36.9%).

Interesting differences were reported for limonene content. In fact, the northern Greek oil showed a content of 27.7%, unlike the oil from southern Greece with a content of 17.1%. Spanish oils showed a percentage of limonene of 4.5%, similar to the percentage we observed in our sample. The greatest differences were found for the bicyclic terpene δ-3-carene, since the oil from northern Greece and Spain had small traces compared to the southern Greek oil, which showed a high percentage content of 13.7%. Of particular interest was the absence of (*E*,*Z*)-farnesol from the Greek and Spanish EOs examined by Adams et al. [9] as well as from those analysed in other countries.

Adams et al. [31], in a comparative study of the phytochemical profiles of the oils extracted from the leaves of *J. oxycedrus*, stated that generally, the oils of this species originating from western Mediterranean countries (Morocco, Spain, France, and Portugal) had a higher content of α-pinene (40–50%) than the oils originating from eastern Mediterranean countries (Italy, Greece, Croatia, Turkey) (20–40%).

Important differences were also present among plants of *J. oxycedrus* collected in different regions of Turkey. In this regard, Hayta and Bagci [14] analysed the essential oils extracted by hydro-distillation from different parts of *J. oxycedrus*, including the leaves.

Data showed also in this case that α-pinene and limonene were the most abundant monoterpenes. *J. oxycedrus* essential oil from Algeria showed *trans*-pinocarveol (7.0%) and *cis*-verbenol (6.3%) as characteristic compounds, not found in such quantities in samples from other Mediterranean regions [13]. In conclusion, *J. oxycedrus* oil from different regions of the Mediterranean is very variable in terms of chemical composition. Surely, this variability is related to the peculiar climatic characteristics that differ from region to region, and to factors related to the type of soil as well as to the altitude where this plant grows.

*J. oxycedrus* and *J. macrocarpa* showed similar flavonoid and phenolic compound fingerprints, whereas they differed in terms of quantitative content. In light of the obtained data, the major identified constituents belonging to the flavonoid class were (−)-epicatechin, rutin, catechin, quercetin, quercetin-3-*O*-glucoside, and luteolin.

The high (−)-epicatechin and quercetin-3-*O*-glucoside contents were previously demonstrated [15,32]. Taviano et al. [33] found high quantities of these compounds also in the fruits of *J. macrocarpa*. Protocatechuic acid was found to be the most abundant, with a content of 3355 μg/g.

There are numerous previous reports on the composition of the EOs, while few studies have investigated the composition of polar extracts of *Juniperus* species. Among them, Yaglioglu et al. [34] confirmed that rutin is one of the main phenolic compounds that characterize the methanol extract of the *J. oxycedrus*, followed by catechin.

In our methanol extract of *J. oxycedrus*, the amount of catechin was significantly less than that of rutin. This result is in disagreement with that reported by Yaglioglu et al. [34]. In fact, in this work, the methanol extract of *J. oxycedrus* showed about twice the catechin content (274.85 mg/g) compared to that of rutin (146.57 mg/g). This divergence was certainly due to differences in the locations where the plants were grown and collected, and, therefore, to climatic and weather differences to which the plants were exposed, as well as to the types of soil in which they grew. Apigenin was more abundant in *J. oxycedrus* extracts than in those from *J. macrocarpa*, in contrast to data obtained by Lesjak et al. [15] that found a content of 1407.21 µg/g.

In our work, *J. oxycedrus* and *J. macrocarpa* extracts showed promising antioxidant effects. Previously, Živić et al. [35] investigated the DPPH radical scavenging activity of different extracts obtained from *J. oxycedrus* berries. The greatest ability to neutralize DPPH radicals was found with ethanol and ethyl acetate extracts, with IC_50_ values of 2.55 and 106.40 μg/mL, respectively, whereas a lower activity was observed with chloroform extract (IC_50_ of 257.66 μg/mL). It is evident that the extracts obtained with more polar solvents possessed higher antioxidant activity due to the high TPC. More recently, the antioxidant potential of *J. phoenicea* subsp. *phoenicea* and *J. oxycedrus* were analysed. In particular, the highest DPPH radical scavenging potential was observed with *J. oxycedrus* fruit essential oil, with an IC_50_ value of 20.2 mg/mL, followed by *J. phoenicea* leaves (36.1 mg/mL). At a concentration of 9.2 mg/mL, all tested essential oils were able to reduce Fe^3+^ to Fe^2+^ [36].

The FRAP ability was confirmed also by Gök et al. [37], who investigated the *J. macrocarpa* branch and leaf ethyl acetate extracts, finding absorbance values of 3.12 and 3.19, respectively at 3 mg/mL, as well as methanol extracts (absorbance values of 3.31 and 3.02 for branch and leaf extracts, respectively). All extracts were characterized by higher absorbance values than that found for positive control ascorbic acid. A FRAP value of 71.50 mg of ascorbic acid equivalents/g of dried weight was recorded for *J. macrocarpa* leaf 80% aqueous methanol extract by Lesjak et al. [15], whereas no FRAP activity was observed with essential oil.

Several *Juniperus* species were found to be able to exert anti-proliferative activity, and different extracts were investigated. The cytotoxic activity of *J. macrocarpa* against the human lung adenocarcinoma cell line was confirmed by Calderón-Montaño et al. [38], who found IC_50_ values of 3.7 and 146.1 mg/mL for ethanol/ethyl acetate/water extracts of the aerial parts and monosperma cones, respectively. Recently, Lai et al. [39] demonstrated that *J. communis* fruit extract exerted promising cytotoxicity activity against colorectal cancer cells with IC_50_ values of 54.32 and 27.3 μg/mL for HT-29 and CT-26 after 72 h of exposure, respectively. Moreover, the phytocomplex demonstrated a synergistic effect when combined with 5-fluorouracil. An in vivo study confirmed that *J. communis* extract induced cell cycle arrest at the G_0_/G_1_ phase via regulation of p53/p21 and CDK4/cyclin D1 and induced cell apoptosis via the extrinsic and intrinsic pathways.

A similar mechanism was observed also with *J. communis* extract against human oral squamous cancer cells (OECM-1) with an IC_50_ value of 45.83 μg/mL after 72 h of exposure [40].

*J. communis* (wild clone) needle EO displayed anti-proliferative activity in a concentration-dependent manner with IC_50_ values of 98.0, 134.4, and 150.6 μg/mL against A431, A549, and SiHa cancer cells, respectively [41].

Yaglioglu et al. [42] investigated the essential oils obtained from the needles and cones of four *Juniperus* species from Turkey—*J. oxycedrus* ssp. *oxycedrus*, *J. foetidissima*, *J. excelsa*, and *J. communis*—for their anti-proliferative activities against HeLa (human cervical carcinoma) and C6 (rat brain tumour) cell lines. The results revealed that, generally, the needles had better anti-proliferative activity than the cones against both cell lines. However, the needles had strong anti-proliferative activities selective against the C6 cells. The essential oil of the needles of *J. excelsa* showed strong anti-proliferative activity against both HeLa and C6 cell lines. Recently, we analysed the anti-proliferative activity of *n*-hexane and dichloromethane extracts of *J. macrocarpa* and *J. oxycedrus* [43]. Our results highlighted the promising activity of *J. oxycedrus*. In fact, both *J. oxycedrus n*-hexane and dichloromethane extracts exhibited cytotoxic activity against COR-L23 cells with IC_50_ values of 26.9 and 39.3 µg/mL, respectively, while *J. macrocarpa* extracts were not active at the highest tested concentration, except for the *n*-hexane extract with an IC_50_ value of 75.1 µg/mL.

## 4. Materials and Methods

### 4.1. Chemicals and Reagents

Solvents of analytical grade used in this study were obtained from VWR International s.r.l. (Milan, Italy). Acetonitrile and water of HPLC grade were purchased from Carlo Erba (Milan, Italy). Ascorbic acid, 2,2′-azino-bis(3-ethylbenzothiazoline-6-sulfonic acid (ABTS) solution, butylated hydroxytoluene (BHT), β-carotene, 2,2-diphenyl-1-picrylhydrazyl (DPPH), Tween 20, linoleic acid, dimethyl sulfoxide (DMSO), trichloroacetic acid (TCA), Dulbecco’s Modified Eagle Medium (DMEM), Roswell Park Memorial Institute (RPMI) 1640 medium, and propyl gallate were purchased from Sigma-Aldrich S.p.a. (Milan, Italy). Apigenin, caffeic acid, (+)-catechin, chlorogenic acid, (−)-epicatechin, gallic acid, kaempferol, kaempferol-3-*O*-glucoside, luteolin, neo-chlorogenic acid, vinblastine sulfate salt, taxol, protocatechuic acid, quercetin, quercetin-3-*O*-glucoside, rutin, syringic acid, and vanillic acid were purchased from Sigma-Aldrich (Milan, Italy).

### 4.2. Plant Materials and Extraction

The leaves of *Juniperus macrocarpa* and *J. oxycedrus* were harvested in March 2016 from plants cultivated in the Botanical Garden, University of Calabria that had been collected in Galatrella valley, Tarsia (CS, Calabria, Italy) (Galatrella, sample number: 354, 1 individual) for *J. oxycedrus*, and in Dune di Sovereto, Isola Capo Rizzuto (KR, Calabria, Italy) (Capo Rizzuto, sample number: 681, 2 individuals), for *J. macrocarpa*. The voucher specimens have been preserved in the herbarium of the Botanical Garden under accession numbers 5853 and 5867 for *Juniperus macrocarpa* and *J. oxycedrus*, respectively.

Fresh leaves of *J. macrocarpa* (480 g) and *J. oxycedrus* (480 g) were steam distilled for 3 h using a clevenger-type apparatus. White-yellow essential oils were obtained. The essential oils were dried over anhydrous sodium sulphate and stored in hermetically sealed brown glass bottles at 4 °C before analyses.

Fresh leaves of *J. macrocarpa* (180 g) and *J. oxycedrus* (180 g) were extracted by exhaustive maceration (700 mL, 4 × 72 h) using ethyl acetate and methanol as solvents. Two extracts for each *Juniperus* species were then obtained after the removal of the extraction solvents at reduced pressure, whose weights were 10.9 and 18.7 g for the ethyl acetate and methanol extracts of *J. macrocarpa*, respectively, and 7.6 and 19.4 g for the ethyl acetate and methanol extracts of *J. oxycedrus*, respectively. Dried extracts were kept in brown bottles at 4 °C before analyses.

### 4.3. Phytochemical Screening

Essential oils (EOs) of *J. macrocarpa* and *J. oxycedrus* were analysed using a Hewlett-Packard 6890 gas chromatograph and a Hewlett-Packard 5973 mass selective detector equipped with an HP-5 MS column (30 m × 0.25 mm i.d., film thickness 0.25 μm) [44]. The oven temperature was isothermally programmed at 50 °C for 5 min, rising from 50 to 250 °C at 13 °C/min, and then held isothermally at 250 °C for 10 min. The carrier gas was helium (1.0 mL/min), ionization of the sample components, EI (70 eV). The tentative identification of the compounds was based on the comparison of their retention indices, in relation to the retention times of a series of *n*-alkanes (C_9_-C_31_), with those of the literature or with those of authentic compounds [45,46]. Further identification was made by comparison of their mass spectra with those stored in the Wiley 138 and NIST98 libraries or with published mass spectra. The GC analyses were performed using a Shimadzu GC17A apparatus controlled by Borwin Software and equipped with a flame ionization detector (FID) and an HP-5 column (30 m, 0.25 mm i.d., film thickness 0.25 μm) (Shimadzu, Milan, Italy). The oven temperature was programmed as reported for GC-MS analyses. The carrier gas was nitrogen (1.0 mL/min). The relative concentrations of the components were calculated based on the GC peak areas without using correction factors.

Ethyl acetate and methanol extracts of *J. macrocarpa* and *J. oxycedrus* were analysed using a Knauer (Asi Advanced Scientific Instruments, Berlin) system equipped with two pumps (Smartline Pump 1000), a Rheodyne injection valve (20 mL), and a photodiode array detector UV/VIS equipped with a semi micro-cell [47]. Clarity Software (Chromatography Station for MS Windows) was used for data processing. A Knauer RP C18 column (250 mm × 4.6 mm, 5 μm) was used. The mobile phase was water/formic acid (99.9:0.1, *v*/*v*) as solvent A, and acetonitrile/formic acid (99.9:0.1, *v*/*v*) as solvent B. The gradient profile was 0.01–20.00 min 5% B isocratic; 20.01–50.00 min, 5–40% B; 50.01–55.00 min, 40–95% B; 55.01–60.00 min 95% B isocratic. The flow rate was 1.0 mL/min. Samples were filtered through a membrane filter with pore size of 0.45 µm (GMF Whatman) before injection took place. The injection volume was 20 μL. Peaks were monitored at 280 and 350 nm.

Apigenin, caffeic acid, (+)-catechin, chlorogenic acid, (−)-epicatechin, gallic acid, kaempferol, kaempferol-3-*O*-glucoside, luteolin, neochlorogenic acid, protocatechuic acid, quercetin, quercetin-3-*O*-glucoside, rutin, syringic acid, and vanillic acid were chosen as reference compounds and quantified. A standard mixture was prepared by adding an accurately weighed amount of each compound (100 mg) to a 100 mL volumetric flask and was brought to the mark with methanol (9:1). A straight calibration for each standard was obtained by analysing the standard solution diluted at different concentrations. All solutions were filtered through a 0.45 μm membrane filter (GMF Whatman) and injected into the HPLC system to determine retention times. Identification and quantification were carried out based on recorded retention times in comparison with authentic standards. Analyses were performed in triplicate.

### 4.4. In Vitro Antioxidant Activity

The antioxidant activity was evaluated by applying four in vitro assays: i.e., the β-carotene bleaching test, ABTS (2,2′-azino-bis(3-ethylbenzothiazoline-6-sulphonic acid) test, DPPH (2,2-diphenil-1-picrylhydrazyl) test, and ferric reducing antioxidant power (FRAP) test.

In the β-carotene bleaching assay, a mixture of linoleic acid, β-carotene, and Tween 20 was prepared and after evaporation of the solvent and dilution with water, the obtained emulsion and *Juniperus* samples were added into tubes that were placed in a water bath at 45 °C [48]. The absorbance was measured at 470 nm at the initial time (t = 0) and after 30 and 60 min of incubation. Propyl gallate was used as a positive control.

In the ABTS test, ABTS radical cation was produced by the reaction of an ABTS solution and potassium persulphate [48]. The obtained solution was diluted with ethanol to an absorbance of 0.70 at 734 nm, and the ABTS scavenging ability was calculated according to the equation: [(A_0_ − A_1_)/A_0_] × 100, where A_0_ is the absorbance of the control reaction and A is the absorbance in the presence of sample. Ascorbic acid was used as positive control.

The DPPH radical scavenging activity was measured at 517 nm, with ascorbic acid used as a positive control [48]. The DPPH radical scavenging activity, expressed as a percentage, was calculated using the formula: [(A_0_ − A_1_)/A_0_] × 100, where A_0_ is the absorbance of the blank and A1 is the absorbance in the presence of the extract.

In the FRAP assay, based on the reaction that involves 2,4,6-tripyridyl-s-triazine)-Fe^3+^ (TPTZ) complex, a mixture of 2.5 mL of 10 mM TPTZ solution in 40 mM HCl, 2.5 mL of 20 mM FeCl_3_, and 25 mL of acetate buffer (0.3 M, pH 3.6) was prepared [49]. The absorbance was measured at 595 nm. Data are expressed as μM Fe(II)/g. Butylated hydroxytoluene (BHT) was used as a positive control.

### 4.5. In Vitro Anti-Proliferative Activity

#### 4.5.1. Cell Lines and Culture Conditions

The cell lines used in this study were human lung adenocarcinoma cell line (A549), human breast cancer ER+ cells (MCF-7, ECACC N°: 86012803), triple negative breast adenocarcinoma cell line (MDA-MB-231, ECACC N°: 92020424), and human Caucasian lung large cell carcinoma (COR-L23, ECACC N°: 92031919). All media, buffers, trypsin, and dyes were filter-sterilized prior to use and warmed to 37 °C. For maintenance purposes, the MDA-MB-231 and CORL-23 cells were cultured in RPMI 1640 medium, while MCF-7 and A549 cells were cultured in DMEM. PC3 and A549 cells were cultured in DMEM/F12 supplemented with 10% foetal bovine serum (FBS), 2 mM L-glutamine, and 1% penicillin/streptomycin. The cell lines were cultured at 37 °C in 5% CO_2_ in a humidified atmosphere. The cultures were passaged once a week by trypsinization using a 1:30 dilution of standard Trypsin-EDTA solution. Cell counts and viability were assessed using a standard trypan blue cell counting technique. All tested samples were dissolved in DMSO and diluted in the appropriate medium to obtain the working concentration.

#### 4.5.2. Cell Viability Assay

Cell viability was determined using the protein-staining sulforhodamine B (SRB) assay as previously described [50]. Briefly, cells were trypsinized, counted, and placed in 96-well plates at optimal plating density of each cell line as determined over a range of 5–15 × 10^4^ to ensure exponential growth throughout the experimental period and to ensure a linear relationship between absorbance at 490 nm and cell number. Cultures were analysed by the SRB assay and incubated to allow for cell attachment.

After 24 h, the cells were treated with serial dilutions of the samples to obtain the final concentrations ranging from 5 to 200 μg/mL for each sample. The final mixture used for treating the cells contained not more than 0.5% of the solvent (DMSO), the same as in the solvent-control wells. After 48 h of exposure, 100 μL of ice-cold 40% trichloroacetic acid (TCA) was added to each of the wells, which were left at 4 °C for 1 h and then washed with distilled water. The TCA-fixed cells were stained for 30 min with 50 μL of 0.4% (*w*/*v*) SRB in 1% acetic acid. Plates were washed with 1% acetic acid and air-dried overnight. For plate reading, the bound dye was solubilised with 100 μL of 10 mM tris base (tris[hydroxymethyl]aminomethane). The absorbance of each well was read on a Molecular Devices SpectraMax Plus Plate Reader (Molecular Devices, Celbio, Milan, Italy) at 490 nm. Cell survival was measured as the percentage absorbance compared to that of the untreated control. Vinblastine sulfate salt and taxol were used as a positive control.

### 4.6. Statistical Analysis

The concentration giving 50% inhibition (IC_50_) was calculated by nonlinear regression with the use of GraphPad Prism version 4.0 for Windows (GraphPad Software, San Diego, CA, USA). The concentration–response curve was obtained by plotting the percentage inhibition versus concentration. Differences within and between groups were evaluated by one-way analysis of variance test (ANOVA) followed by a multiple comparison Dunnett’s test compared with the positive control. Data on oil chemical profiles of *J. oxycedrus* were retrieved from the literature (Table 2). A first data matrix was built with 68 cases and 248 oils. We excluded cases when oil extraction was performed on fruits (two cases) or on material collected in the autumn/winter season (10 cases).

Furthermore, we excluded outliers by visual inspections of nonmetric multidimensional scaling, limiting cases to the core variability of *J. oxycedrus*, *J. macrocarpa*, and *J. deltoides*, and excluding cases widespread in the plot, resulting in a total of 40 cases. In our analysis, only oils that were present in at least 80% of cases for each species were included, resulting in a total of 30 oils. The final data matrix (40 cases × 30 oils) was subjected to multivariate analysis. Ordination was performed through principal coordinate analysis using the chord distance because of the frequent occurrence of double zero. Classification was performed using the unweighted pair group method with arithmetic mean (UPGMA) agglomerative method on chord distance. Silhouette analysis was used to obtain the best number of clusters (Silhouette score) and the relative significance (average distance), and an average distance over 0.5 was considered significant.

## 5. Conclusions

In recent decades, thanks to the growing number of studies on medicinal plants, their phytochemicals, and their potential applications, interest in natural products has increased. In this context, *Juniperus* species have been extensively studied and demonstrated the presence of a wide array of compounds with a variety of biological effects. Herein, essential oils and polar extracts of *J. oxycedrus* and *J. macrocarpa* aerial parts collected in southern Italy were subjected to a comparative study of their chemical profiles and anti-proliferative and antioxidant properties.

Previous studies described the anti-proliferative effects of some *Juniperus* species on several human cancer cell lines including lung cancer cells (A549) and breast cancer cells (MCF-7).

To the best of our knowledge, this is the first report highlighting the anti-proliferative activity of *J. oxycedrus* and *J. macrocarpa* methanol and ethyl acetate extracts against triple negative breast adenocarcinoma (MDA-MB-231) and human lung large cell carcinoma (COR-L23).

The most promising activities were evidenced with *J. oxycedrus* methanol and ethyl acetate extracts, which exerted good radical scavenging activity and exhibited remarkable activity against the large lung carcinoma cell line (COR-L23) with IC_50_ values of 26.0 and 39.1 μg/mL, respectively, lower than that obtained with positive control vinblastine with an IC_50_ value of 45.5 μg/mL. The World Health Organization (WHO) indicates that lung cancer is the most common type of cancer, with 2.09 million cases in 2019 [51]. Many natural compounds could specifically target different cell signalling pathways associated with cancer progression to provide a cytotoxic effect in the target cell. The importance of these compounds is emerging in many therapies developed with dual action often including a natural compound. Currently, there are many natural compounds or their derivatives in combination with synthetic drugs for lung cancer at different stages of clinical trials. The results of this study indicate that *J. oxycedrus* may be considered a source of natural compounds with antioxidant and anti-proliferative effects that could be suitable for future applications.

## Figures and Tables

**Table 1:** The main identified constituents of *J. macrocarpa* and *J. oxycedrus* essential oils.

Compound	Class	RI ^a^	%		I.M ^b^	Sign
			*J. macrocarpa*	*J. oxycedrus*		
Tricyclene	mh	928	0.2 ± 0.02	*n.d.*	1,2	**
α-Pinene	mh	938	25.3 ± 2.5	36.9 ± 2.5	1,2,3	**
Camphene	mh	953	0.6 ± 0.04	0.6 ± 0.03	1,2,3	ns
β-Pinene	mh	980	2.6 ± 0.9	5.5 ± 0.9	1,2,3	**
Myrcene	mh	993	3.1 ± 0.7	2.7 ± 0.1	1,2,3	**
Sabinene	mh	973	8.2 ± 1.1	0.7 ± 0.03	1,2,3	**
α-Phellandrene	mh	1005	3.3 ± 0.8	1.1 ± 0.04	1,2	**
δ-3-Carene	mh	1009	2.4 ± 0.02	0.6 ± 0.03	1,2	**
α-Terpinene	mh	1012	0.7 ± 0.05	1.9 ± 0.5	1,2,3	**
*p*-Cymene	mh	1025	3.2 ± 1.1	0.5 ± 0.04	1,2	**
Limonene	mh	1030	3.4 ± 0.03	6.3 ± 0.9	1,2,3	**
(*E*)-β-Ocimene	mh	1048	2.8 ± 0.7	0.2 ± 0.01	1,2	**
γ-Terpinene	mh	1057	0.5 ± 0.01	0.2 ± 0.01	1,2,3	ns
Terpinolene	mh	1086	1.5 ± 0.7	0.3 ± 0.02	1,2,3	**
Nonanal	oc	1102	0.1 ± 0.01	0.3 ± 0.01	1,2	*
α-Campholene aldehyde	om	1132	1.2 ± 0.6	0.5 ± 0.02	1,2	**
Camphor	om	1145	0.3 ± 0.03	tr	1,2	**
*p*-Mentha-1,5-dien-8-ol	om	1167	0.4 ± 0.04	*n.d.*	1,2	**
Terpinen-4-ol	om	1176	0.3 ± 0.02	tr	1,2	**
α-Terpineol	om	1189	2.9 ± 0.9	0.3 ± 0.02	1,2,3	**
Myrtenal	om	1196	*n.d.*	0.2 ± 0.01	1,2	**
Decanal	oc	1205	0.2 ± 0.02	0.3 ± 0.03	1,2	ns
Verbenone	om	1206	0.5 ± 0.05	*n.d.*	1,2	**
(−)-Carvone	om	1242	0.2 ± 0.03	*n.d.*	1,2	*
Piperitone	om	1254	0.2 ± 0.02	*n.d.*	1,2	*
Phellandral	om	1281	0.3 ± 0.02	0.2 ± 0.01	1,2	ns
(−)-Bornyl acetate	om	1286	0.2 ± 0.01	*n.d.*	1,2	*
α-Cubebene	sh	1352	0.2 ± 0.01	0.5 ± 0.01	1,2	ns
α-Copaene	sh	1377	0.2 ± 0.02	0.3 ± 0.02	1,2	ns
β-Bourbonene	sh	1385	0.3 ± 0.02	0.9 ± 0.02	1,2	**
β-Cubebene	sh	1387	1.8 ± 0.6	*n.d.*	1,2	**
α-Gurjunene	sh	1407	*n.d.*	1.1 ± 0.04	1,2	**
*trans*-Caryophyllene	sh	1415	0.2 ± 0.01	2.0 ± 0.2	1,2,3	**
*trans*-α-Bergamotene	sh	1438	*n.d.*	0.4 ± 0.01	1,2	**
α-Humulene	sh	1455	0.2 ± 0.01	1.5 ± 0.08	1,2	**
Germacrene D	sh	1477	4.5 ± 0.03	2.0 ± 0.07	1,2	**
γ-Cadinene	sh	1515	0.2 ± 0.01	5.4 ± 0.6	1,2	**
δ-Cadinene	sh	1526	0.4 ± 0.01	2.7 ± 0.6	1,2	**
(*E*)-β-Farnesene	sh	1452	0.4 ± 0.02	0.7 ± 0.01	1,2	**
α-Muurolene	sh	1500	0.3 ± 0.01	0.4 ± 0.01	1,2	ns
Caryophyllene oxide	os	1580	0.9 ± 0.04	3.8 ± 0.5	1,2	**
(*Z*,*E*)-Farnesol	os	1722	2.8 ± 0.2	6.5 ± 0.8	1,2,3	**
Manoyl oxide	di	1989	6.6 ± 0.6	2.4 ± 0.4	1,2	**
13-*epi*-Manoyl oxide	di	1994	0.6 ± 0.2	0.6 ± 0.02	1,2	ns
(*Z*)-Phytol	di	1950	*n.d.*	0.5 ± 0.01	1,2	**
Abietatriene	di	2054	2.9 ± 0.7	2.6 ± 0.3	1,2	*
Abietadiene	di	2080	1.8 ± 0.6	1.7 ± 0.3	1,2	ns
Heneicosane	oc	2100	*n.d.*	0.2 ± 0.02	1,2,3	*
Tricosane	oc	2300	0.3 ± 0.02	0.2 ± 0.01	1,2,3	ns
Pentacosane	oc	2500	0.2 ± 0.01	0.5 ± 0.01	1,2,3	*
Heptacosane	oc	2700	0.4 ± 0.01	0.5 ± 0.02	1,2,3	ns
Nonacosane	oc	2900	0.4 ± 0.03	0.3 ± 0.01	1,2,3	ns
Monoterpene hydrocarbons	mh		57.8	57.5		
Oxygenated monoterpens	om		6.5	1.2		
Sesquiterpene hydrocarbons	sh		8.7	17.9		
Oxygenated sesquiterpenes	os		3.7	10.3		
Diterpenes	di		11.9	7.8		
Other constituents	oc		1.6	2.3		
Total			90.2	97.0		

Data are expressed as mean ± standard deviation (S.D.) (*n* = 3). *n.d*.: not detected. tr: trace (<0.1%). ^a^ Retention Index (RI) on HP-5 MS column. ^b^ IM: identification methods: 1—comparison of retention times; 2—comparison of mass spectra with MS libraries; 3—comparison with authentic compounds. Differences were evaluated by one-way analysis of variance (ANOVA) completed with a multiple comparison Tukey’s test (** *p* < 0.01, * *p* < 0.05). ns: not significant.

**Table 2:** HPLC-DAD profiles (μg/g) of *J. macrocarpa* and *J. oxycedrus* polar extracts.

	*J. macrocarpa*		*J. oxycedrus*		
Compound	Ethyl Acetate Extract	Methanol Extract	Ethyl Acetate Extract	Methanol Extract	Sign
Apigenin	41.6 ± 1.7 ^dN^	82.7 ± 3.6 ^cM^	243.6 ± 5.3 ^bC^	324.8 ± 8.2 ^aF^	**
Caffeic acid	43.5 ± 1.2 ^aM^	31.4 ± 2.1 ^bP^	19.3 ± 4.3 ^Cm^	10.7 ± 0.2 ^dN^	**
(+)-Catechin	645.4 ± 5.6 ^bD^	915.5 ± 2.1 ^aC^	108.4 ± 7.7 ^dF^	537.0 ± 5.4 ^cD^	**
Chlorogenic acid	313.6 ± 2.5 ^aE^	141.3 ± 6.8 ^cH^	45.8 ± 6.2 ^dI^	246.2 ± 9.2 ^bG^	**
(−)-Epicatechin	161.0 ± 1.0 ^dF^	211.4 ± 4.6 ^cE^	4237.6 ± 5.7 ^aA^	3874.5 ± 4.2 ^bB^	**
Gallic acid	713.7 ± 6.6 ^aC^	684.3 ± 8.8 ^bD^	0 ^cQ^	0 ^cQ^	**
Kaempferol	35.4 ± 0.9 ^bO^	10.8 ± 4.5 ^dR^	15.6 ± 1.3 ^cN^	48.6 ± 3.5 ^aI^	**
Kaempferol-3-*O*-glucoside	66.2 ± 2.2 ^bL^	189.3 ± 7.3 ^aF^	2.7 ± 0.1 ^dP^	8.6 ± 0.8 ^cO^	**
Luteolin	10.1 ± 0.1 ^dQ^	78.5 ± 3.3 ^cN^	155.7 ± 8.4 ^bE^	329.6 ± 8.7 ^aE^	**
Neochlorogenic acid	34.0 ± 1.3 ^cO^	130.9 ± 4.2 ^aL^	28.5 ± 0.9 ^dL^	40.5 ± 1.5 ^bL^	**
Protocatechuic acid	1091.0 ± 7.2 ^bB^	1142.0 ± 9.2 ^aB^	0 ^cQ^	0 ^cP^	**
Quercetin	137.2 ± 5.3 ^cH^	133.6 ± 5.3 ^dI^	192.4 ± 10.1 ^bD^	201.5 ± 5.5 ^aH^	**
Quercetin-3-*O*-glucoside	1533.4 ± 9.12 ^cA^	1769.5 ± 4.3 ^bA^	2937.3 ± 5.6 ^aB^	1404.5 ± 7.2 ^dC^	**
Rutin	149.3 ± 5.5 ^cG^	168.4 ± 3.8 ^bG^	65.6 ± 4.2 ^dH^	4016.4 ± 3.8 ^aA^	**
Syringic acid	24.0 ± 1.2 ^bP^	21.1 ± 2.0 ^cQ^	13.5 ± 0.8 ^dO^	26.71 ± 0.4 ^aM^	**
Vanillic acid	85.4 ± 15.3 ^aI^	57.4 ± 2.8 ^dO^	71.4 ± 5.7 ^bG^	65.3 ± 0.8 ^cH^	**
Sign	**	**	**	**	

Data are expressed as mean ± standard deviation (S.D.) (*n* = 3). Differences were evaluated by one-way analysis of variance (ANOVA) completed with a multiple comparison Tukey’s test; ** *p* < 0.05. Differences were evaluated by one-way analysis of variance (ANOVA) completed with a multiple comparison Tukey’s test; ** *p* < 0.05. Means in the same row with different small letters differ significantly between samples (*p* < 0.05), while capital letters differ significantly between compounds.

**Table 3:** Antioxidant activity of essential oils and extracts of *J. macrocarpa* and *J. oxycedrus*.

Sample	ABTS IC_50_ (μg/mL)	DPPH IC_50_ (μg/mL)	FRAP Test μM Fe(II)/g ^c^	β-Carotene Bleaching Test IC_50_ (μg/mL)	
				30 min	60 min
*J. macrocarpa*					
Essential oil	20.4% ^a^	34.1% ^b^	2.4 ± 0.2	54.8 ± 3.4	49.4 ± 2.8
Ethyl acetate extract	147.6 ± 4.8	40.9 ± 2.4	26.4 ± 1.8	84.9 ± 3.5	95.7 ± 3.9
Methanol extract	39.1 ± 1.7	29.3 ± 1.5	23.6 ± 1.5	65.1 ± 2.2	62.5 ± 2.8
*J. oxycedrus*					
Essential oil	5.2% ^a^	31.6% ^b^	3.8 ± 0.3	47.5 ± 2.6	5.9 ± 3.4
Ethyl acetate extract	9.3 ± 1.3	20.6 ± 2.3	99.5 ± 3.7	15.1 ± 1.1	13.2 ± 0.8
Methanol extract	6.2 ± 1.1	19.7 ± 2.5	101.9 ± 3.9	23.1 ± 1.2	17.1 ± 0.9
Positive control					
Ascorbic acid	1.7 ± 0.4	5.1 ± 0.8			
BHT			63.2 ± 4.4		
Propyl gallate				1.1 ± 0.05	1.0 ± 0.06

Data are expressed as mean ± SD (*n* = 3). ^a^ at the maximum concentration tested (500 μg/mL). ^b^ at the maximum concentration tested (1000 μg/mL). ^c^ at 2.5 mg/mL.

**Table 4:** Anti-proliferative activity (IC_50_ μg/mL) of essential oils and extracts of *J. macrocarpa* and *J. oxycedrus* against four cancer cell lines (MCF-7, MDA-MB-231, A549, and COR-L23).

Sample	MCF-7	MDA-MB-231	A549	COR-L23
*J. macrocarpa*				
Essential oil	85.4 ± 3.2 **	96.4 ± 3.8 **	>200	101.0 ± 3.9 **
Ethyl acetate extract	163.4 ± 4.9 **	186.2 ± 5.1 **	>200	>200
Methanol extract	>200	>200	>200	>200
*J. oxycedrus*				
Essential oil	>200	>200	>200	>200
Ethyl acetate extract	147.9 ± 4.6 **	158.1 ± 5.1 **	>200	39.1 ± 1.4 **
Methanol extract	>200	>200	87.9 ± 4.7 **	26.0 ± 1.3 **
*Positive control*				
Taxol	0.08 ± 0.004	1.6 ± 0.03		
Vinblastine sulfate			67.3 ± 2.0	45.5 ± 0.7

Data are expressed as median ± S.D. (*n* = 3). A549: human lung adenocarcinoma cell line; MCF-7: human breast cancer ER+ cell line; MDA-MB-231: triple negative breast adenocarcinoma cell line; COR-L23: human lung large cell carcinoma cell line. ** *p* < 0.01 vs. positive control.

**Table 5:** The dominant volatiles of *J. macrocarpa* and *J. oxycedrus* essential oils from data in the literature.

Compounds	Origin	Ref.
*J. macrocarpa*		
α-Pinene (25.3%), *p*-cimene (13.2%), sabinene (8.2%)	Italy	Our data
Manoyl oxide (7.7–21.9%), α-pinene (7.2–11.1%), α-cedrol (2.3–9.7%)	Turkey	[7]
Sabinene (26.5%), α-pinene (22.6%), terpinen-4-ol (7.3%)	Spain	[9]
Gemacrene D (21.3%), (*Z*,*E*)-farnesol (10.9%), 8,13-epoxy-14,15-dinorlabdane (8.8%)	Algeria	[12]
α-Pinene (49.4%), gemacrene D (18.1%), β-phellandrene (3.8%)	Croatia	[15]
α-Pinene (15.9%), sabinene (12.1%), δ-3-carene (5.9%)	Tunisia	[16]
α-Pinene (22.8%), sabinene (9.1%), *p*-cimene (7.3%)	Tunisia	[16]
α-Pinene (26.9%), cedrolo (13.9%), dihydro-*p*-cimen-8-ol (8.5%)	Greece	[17]
α-Pinene (22.8%), α-terpineol (18.7%), 1,8-cineole (9.1%)	Italy	[18]
α-Pinene (81.3%), γ-muurolene (2.6%), β-pinene (2.1%)	Italy	[18]
α-Pinene (73.5%), α-terpineol (3.3%), β-pinene (2.1%)	Italy	[18]
α-Pinene (58.0%), cedrol (7.3%), α-muurolene (2.4%)	Greece	[19]
*J. oxycedrus*		
α-Pinene (36.9%), limonene (6.3%), (*Z*,*E*)-farnesol (6.5%)	Italy	Our data
α-Pinene (17.1%), 13-*epi*-manoyl oxide (12.5%), (*Z*)-6-pentadecen-2-one (11.5%)	Morocco	[8]
α-Pinene (41.3%), α-phellandrene (8.2%), *p*-cymene (6.2%)	Spain	[9]
Limonene (27.7%), α-pinene (25.3%), myrcene (3.8%)	Greece	[9]
α-Pinene (42.7%), limonene (17.1%), δ-3-carene (13.7%)	Greece	[9]
Manoyl oxide (32.8%), caryophyllene oxide (11.9%), germacrene D (5.7%)	Turkey	[10]
α-Pinene (31.2%), sabinene (5.2%), limonene (5.0%)	Morocco	[11]
*trans*-Pinocarveol (7.0%), *cis*-verbenol (6.3%), manoyl oxide (6.0%)	Algeria	[13]
α-Pinene (42.9%), limonene (17.8%), caryophyllene oxide (5.1%)	Turkey	[14]
α-Pinene (49.5%), germacrene D (8.9%), 13-*epi*-manoil ossido (3.6%)	Tunisia	[16]
Limonene (30.0%), α-pinene (26.3%), (*Z*,*E*)-farnesol (5.1%)	Italy	[18]
